# Plasma Mitochondrial DNA and Necroptosis as Prognostic Indicators in Critically Ill Patients with Sepsis

**DOI:** 10.3390/biomedicines10102386

**Published:** 2022-09-25

**Authors:** Hayoung Choi, Hongseok Yoo, Jin Young Lee, Junseon Park, Kyeongman Jeon

**Affiliations:** 1 Division of Pulmonary, Allergy, and Critical Care Medicine, Department of Internal Medicine, Hallym University Kangnam Sacred Heart Hospital, Hallym University College of Medicine, Seoul 07441, Korea; 2 Division of Pulmonary and Critical Care Medicine, Department of Medicine, Samsung Medical Center, Sungkyunkwan University School of Medicine, Seoul 06351, Korea; 3 Department of Critical Care Medicine, Samsung Medical Center, Sungkyunkwan University School of Medicine, Seoul 06351, Korea; 4 Department of Health Sciences and Technology, SAIHST, Sungkyunkawan University, Seoul 06351, Korea

**Keywords:** sepsis, biomarker, mitochondrial DNA, necroptosis, damage-associated molecular patterns

## Abstract

Mitochondrial DNA (mtDNA) has been identified as a biomarker for predicting sepsis mortality. Although preclinical studies suggested that necroptosis could explain the mechanistic link of mtDNA in sepsis, this is not yet evident in patients with sepsis. This study evaluated the association between mtDNA and essential necroptosis mediators in prospectively enrolled patients with sepsis. Plasma mtDNA copy number was measured using quantitative PCR assay and necroptosis mediators, including receptor-interacting protein kinase-3 (RIPK3), mixed lineage domain-like pseudokinase (MLKL), and high-mobility group box 1 (HMGB1), were measured by ELISA. Receiver operating characteristic (ROC) analysis was conducted to evaluate the predictive ability of mtDNA copy number as a predictor of hospital mortality. Among the 142 patients with sepsis, the mtDNA copy number was significantly higher in non-survivors than in survivors (median, 4040 copies/µL vs. 2585 copies/µL; *p* < 0.001), and the area under the ROC curve was 0.73 (95% CI, 0.64–0.82) for the relationship between mtDNA and hospital mortality. Furthermore, the correlation between mtDNA copy number and each necroptosis mediator was excellent (*p* < 0.001 for all): RIPK3 (r = 0.803), MLKL (r = 0.897), and HMGB1 (r = 0.603). The plasma mtDNA copy number was highly correlated with essential necroptosis mediators, suggesting that mtDNA propagates necroptosis and increases sepsis mortality.

## 1. Introduction

Sepsis has been identified as a life-threatening organ dysfunction caused by a dysregulated host response to infection [1]. Sepsis and septic shock are major healthcare problems, affecting millions of people worldwide each year and killing approximately 17–33% of affected patients [2,3]. Considering the high mortality in sepsis, accurately identifying high-risk patients with sepsis and focusing treatment on this population is deemed crucial. In this regard, there have been many biomarkers identified for predicting morbidity and mortality in sepsis in the critical care setting [4,5].

Plasma mitochondrial DNA (mtDNA) was assessed as a biomarker for predicting sepsis mortality [6]. Previous studies revealed that plasma mtDNA copy number is significantly higher in patients with sepsis or septic shock than in healthy controls [7,8,9]. Furthermore, mtDNA copy number has also been associated with mortality in patients with sepsis [9,10]. However, increasing evidence has shown that mtDNA is not only a marker for sepsis mortality but also a key player in organ dysfunction and mortality in patients with sepsis. This is because circulating mtDNA can trigger innate immunity through multiple mechanisms, such as activating the Toll-like receptor 9/NF-κB pathway or the NALP3 inflammasome [11,12]. Consequently, mtDNA may contribute to the persistent and dysregulated inflammatory response and amplify organ dysfunction in sepsis [10]. mtDNA serves as damage-associated molecular patterns (DAMPs) that drive an immune or inflammatory response in sepsis [6]. The DAMPs are often released during extensive, infection-induced immune cell death, which is linked with multiple organ dysfunction [13]. 

**Figure 1:**
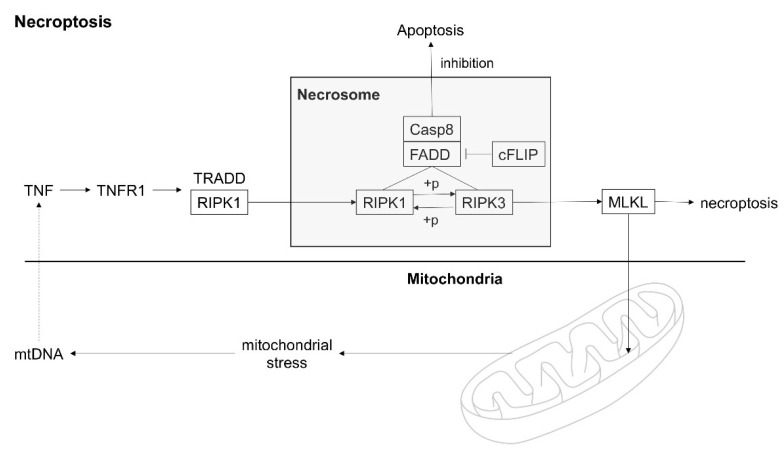
Schematic representation of a correlation between necroptosis and extracellular mitochondrial DNA. TNF, tumor necrosis factor; TNFR1, TNF receptor 1; TRADD, TNFR1-associated death domain; RIPK1, receptor-interacting protein kinase 1; RIPK3, receptor-interacting protein kinase 3; FADD, Fas-associated death domain; Casp8, caspase-8; cFLIP, cellular FLICE (FADD-like IL-1β-converting enzyme)-inhibitory protein; MLKL, mixed-lineage kinase domain-like; mtDNA, mitochondrial DNA.

Necroptosis is a type of cell death that combines apoptosis and necrosis. It is morphologically similar to necrosis, but it can also be regulated by underlying genetic programs [14]. Previous studies suggested that the necroptotic pathway and its regulatory proteins are crucial mediators of sepsis-induced organ injury [15,16,17]. Although a mouse study showed that mtDNA was translocated to the extracellular space by necroptosis [18], clinical studies exploring the association between mtDNA and necroptosis remain to be limited. Figure 1 schematically depicts the correlation between necroptosis and extracellular mtDNA.

This study aimed to evaluate the usefulness of mtDNA as a biomarker to predict mortality in critically ill patients with sepsis and to assess the association between plasma mtDNA levels and necroptosis mediators.

## 2. Materials and Methods

### 2.1. Study Design and Population

The Samsung Medical Center Registry of Critical Illness (SMC-RoCI) is a prospective observational study conducted at the Samsung Medical Center (1989-bed, university-affiliated, tertiary referral hospital in Seoul, South Korea) [16]. This ongoing prospective study was initiated in April 2014 and aims to establish a human sample repository and develop new biological markers for critical illness [16]. The cohort profile was described previously [15,16,17,19]. This study was approved by the Institutional Review Board of Samsung Medical Center, and written informed consent was obtained from all patients or their legally authorized representatives prior to enrollment.

All consecutive adult patients (aged ≥ 19 years) admitted to the medical intensive care unit (ICU) were eligible for inclusion in this registry. The exclusion criteria from eligibility were as follows: (1) cognitive impairment, (2) inability to provide informed consent, (3) ICU admission for a simple procedure or postsurgical care, (4) transfer from other hospitals, (5) end-of-life decision or admission to facilitate palliative care, (6) hemoglobin < 8 g/dL upon admission or persistent bleeding, and (7) discharge within 24 h of ICU admission [16]. 

**Figure 2:**
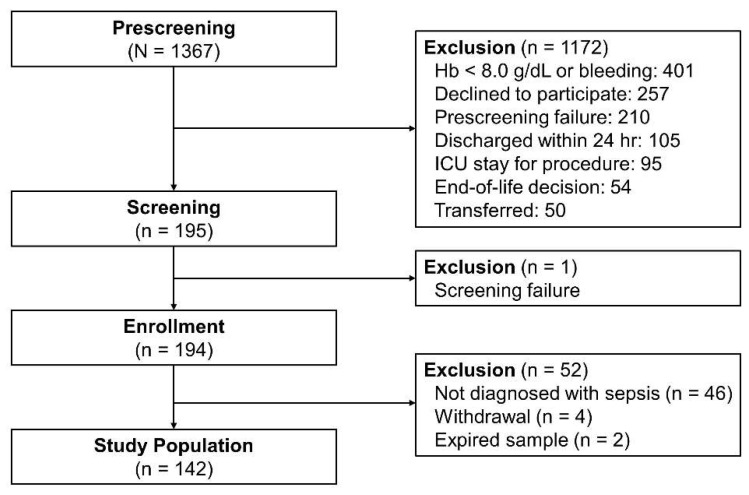
Study flowchart.

In total, 1367 patients were admitted to the medical ICU from April 2014 to August 2016. After excluding 1172 patients who met the exclusion criteria and 1 who missed screening (transferred to the general ward before the screening process), 194 were initially identified. After further excluding 46 patients who were not diagnosed with sepsis, 4 who withdrew consent, and 2 whose samples expired, 142 were ultimately included in the analysis (Figure 2).

### 2.2. Data Collection

Clinical data on patient demographics, the reason for ICU admission, the severity of illness score, and laboratory data were obtained at the time of enrollment. Illness severity was assessed using the Acute Physiology and Chronic Health Evaluation II (APACHE II) [20], Simplified Acute Physiology Score 3 (SAPS 3) [21], and Sequential Organ Failure Assessment (SOFA) scores [22]. Sepsis was defined according to the third International Consensus Definitions for Sepsis and Septic Shock (Sepsis-3) [1]. Since the SMC-RoCI was initiated in April 2014, patients enrolled before the release of the new definition were reclassified using Sepsis-3. 

### 2.3. Measurement of Plasma Mitochondrial DNA and Necroptosis-Related Markers

Blood samples were collected as 19 mL of whole blood within 48 h of study enrollment and centrifuged within 4 h of collection. Plasma was separated and stored at −80 °C for further analysis. In order to measure the mtDNA copy number, the plasma samples were pre-filtered using 0.8 µm syringe filters, and additional centrifugation was performed to eliminate residual cellular material. DNA was isolated from 1 mL of plasma using the QIAamp Circulating Nucleic Acids kit (QIAGEN, Germany) and eluted in 50 µL of kit elution buffer. The mtDNA copy numbers were then assessed by measuring the NADH dehydrogenase 1 gene using quantitative real-time PCR with a PRISM 7300 sequence detection system (Applied Biosystems, Austin, TX, USA) with the following primers: human NADH dehydrogenase 1 gene (mtDNA): forward 5′-ATACCCATGGCCAACCTCCT-3′, reverse 5′-GGGCCTTTGCGTAGTTGTAT-3′; and human β-globin (nuclear DNA): forward 5′-GTGCACCTGACTCCTGAGGAGA-3′, reverse 5′-CCTTGATACCAACCTGCCCAG-3′ [23]. The thermal profile for detecting mtDNA was as follows: denaturation at 95 °C for 10 min, followed by 40 cycles of 10 s at 95 °C, 10 s at 58 °C, and 10 s at 72 °C. In each application, the samples were analyzed in duplicate, and the mean was used in the subsequent analysis.

The plasma levels of necroptosis-related markers (receptor-interacting protein kinase 3 [RIPK3], mixed lineage domain-like [MLKL], and high-mobility group box 1 [HMGB1]) were measured from stored aliquots using commercially available ELISA kits according to the manufacturer’s recommendations (RIPK3; CUSABIO, Wuhan, China) (MLKL; LifeSpan BioSciences, Seattle, DC, USA) (HMGB1; IBL-International, Hamburg, Germany) [15]. 

### 2.4. Statistical Analysis

Categorical variables were compared using the Chi-square test or Fisher’s exact test, whereas continuous variables were compared using the Mann–Whitney *U* test. The receiver operating characteristic (ROC) curve was used to analyze the diagnostic accuracy of mtDNA in predicting survival among critically ill patients with sepsis. Pearson’s correlation analysis was used to assess the association between plasma mtDNA copy numbers and necroptosis-related markers. All tests were two-sided, and *p* < 0.05 was considered significant. Data were analyzed using STATA version 16 (Stata Corp., College Station, TX, USA).

## 3. Results

### 3.1. Baseline Characteristics 

Of the 142 critically ill patients with sepsis, 95 (66.9%) were male, and the median age of all patients was 67 years (interquartile range [IQR], 53–74 years). No significant intergroup differences were found in terms of age, sex, and comorbidities between non-survivors and survivors. Non-survivors received mechanical ventilation therapy more frequently than survivors (55.6% vs. 34.9%, *p* = 0.029). Although no significant intergroup differences were observed in C-reactive protein (CRP) and lactate, non-survivors showed higher SAPS 3 (median, 57 vs. 52; *p* = 0.042) and APACHE II (median, 26 vs. 22; *p* = 0.004) scores than survivors (Table 1). 

### 3.2. Plasma Mitochondrial DNA

**Figure 3:**
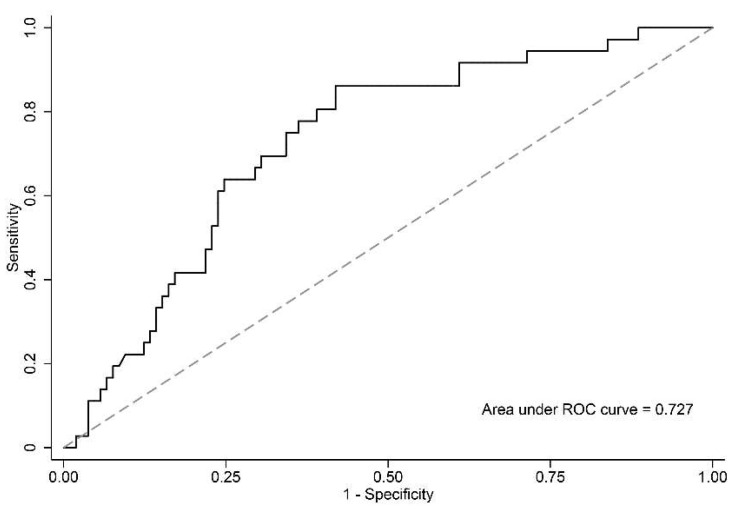
Receiver operating characteristic curve measuring the usefulness of mitochondrial DNA in predicting in-hospital mortality in critically ill patients with sepsis. The area under the curve value was 0.727 (95% confidence interval, 0.635–0.818).

The plasma mtDNA copy number was significantly higher (*p* < 0.001) in non-survivors (median, 4040 copies/µL; IQR, 3232–6288 copies/µL) than in survivors (median, 2585 copies/µL; IQR, 1867–3642 copies/µL) (Table 2). Figure 3 depicts the receiver operating curve of mtDNA for predicting in-hospital mortality of critically ill patients with sepsis. The area under the curve value was 0.727 (95% confidence interval, 0.635–0.818).

### 3.3. Association between Plasma Mitochondrial DNA and Necroptosis-Related Markers

**Figure 4:**
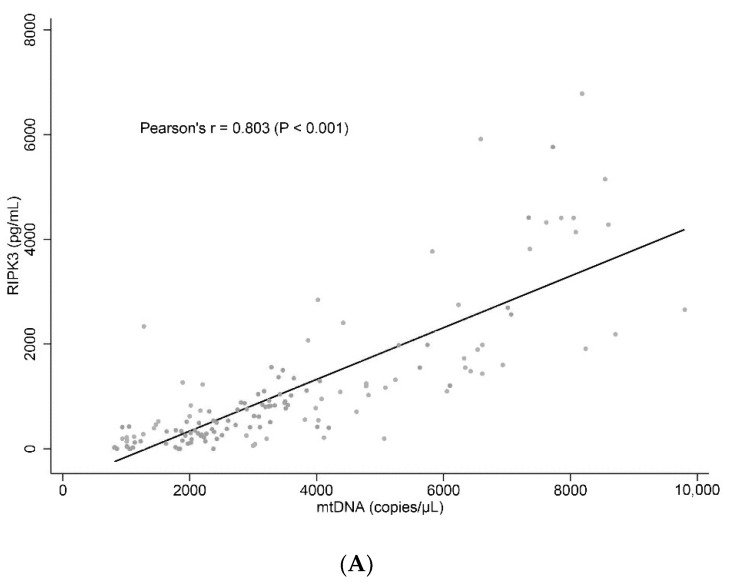

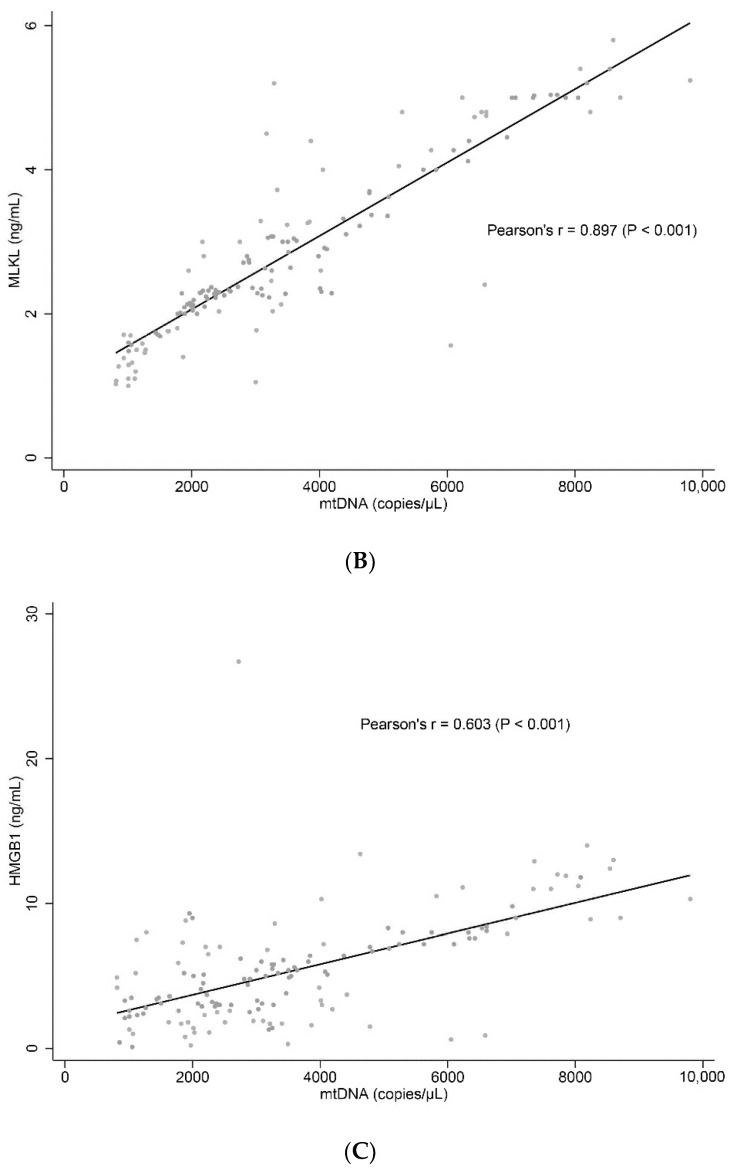
Correlations between mitochondrial DNA and necroptosis-related markers in critically ill patients with sepsis. (**A**) RIPK3, (**B**) MLKL, and (**C**) HMGB1. mtDNA, mitochondrial DNA; RIPK3, receptor-interacting protein kinase-3; MLKL, mixed lineage kinase domain-like; HMGB1, high-mobility group box 1.

The plasma levels of necroptosis-related markers were found to be significantly higher in non-survivors than in survivors (Table 2): RIPK3 (median, 1104 vs. 536 pg/mL; *p* = 0.002), MLKL (median, 3.2 vs. 2.3 ng/mL; *p* = 0.004), and HMGB1 (median, 6.8 vs. 4.5 pg/mL; *p* = 0.017). The correlation between plasma mtDNA copy numbers and each necroptosis-related marker was as follows: mtDNA and RIPK3 (Pearson’s r = 0.803), MLKL (r = 0.897), and HMGB1 (r = 0.603) (*p* < 0.001 for all) (Figure 4).

## 4. Discussion

In this observational study, plasma mtDNA satisfactorily predicted in-hospital mortality in critically ill patients with sepsis. Furthermore, the mtDNA copy number showed excellent correlations with those of necroptosis mediators, including RIPK3, MLKL, and HMGB1, which implies a link between plasma mtDNA and necroptosis in sepsis. 

This study revealed that the mtDNA copy number was significantly higher in non-survivors than in survivors among critically ill patients with sepsis, although no intergroup differences were noted in CRP and lactate levels. In agreement with our results, previous studies found that the mtDNA copy number was elevated in patients with sepsis [7,8,9] and was associated with mortality [9,10]. In this regard, plasma mtDNA may be a suitable biomarker for diagnosing sepsis and predicting mortality in patients with sepsis. However, mtDNA copy number was not significantly associated with mortality in some sepsis studies [8,24]. This discrepancy might be explained by differences in terms of the protocols used to measure mtDNA copy numbers across previous studies, thus warranting the standardization of mtDNA protocols to firmly establish the clinical usefulness of mtDNA in sepsis [25].

In order to serve as a DAMP driving the inflammatory response in sepsis, mtDNA needs to be released from the mitochondria into the cytosol and extracellular compartments [6]. The underlying mechanisms of the phenomenon include the generation of mitochondrial reactive oxygen species [26,27] and destabilized mtDNA packaging [28]. In addition to these two potential mechanisms, necroptosis can translocate mtDNA to the extracellular space, which was then suggested in a mouse study [18]. Excellent correlations between mtDNA level and necroptosis mediators (RIPK3, MLKL, and HMGB1) in this study show that a large proportion of plasma mtDNA may be released by necroptosis in sepsis.

Necroptosis is often induced by specific ligand binding, including the binding of tumor necrosis factor (TNF) and TNF-related apoptosis-inducing ligand (TRAIL) to several death receptors, including TNF receptor 1 and TRAIL receptors [29]. Subsequently, the necroptosis pathway is regulated by distinct proteins, such as RIPK1 and RIPK3, and downstream substrate MLKL [14,30]. The ruptured, dying cell induces the release of several necrosis-associated DAMPs, including HMGB1 [31,32]. Previous animal and clinical studies demonstrated the crucial role of necroptosis in driving mortality during sepsis [15,17,33,34]. In the same vein, this study also showed that plasma RIPK3, MLKL, and HMGB1 levels were significantly higher in non-survivors than in survivors among critically ill patients with sepsis. Furthermore, considering the correlation between plasma mtDNA and necroptosis mediators, mtDNA as a DAMP may propagate a proinflammatory response and consequent necroptosis pathway activation, which may be followed by mortality in patients with sepsis. Overall, mtDNA and necroptosis affect each other, and these interactions could increase mortality in patients with sepsis.

This study has several limitations that should be acknowledged. First, although excellent correlations were observed between plasma mtDNA copy number and necroptosis mediators, this study could not evaluate the causal relationships among them. Future studies are thus warranted to elucidate the mechanistic link between mtDNA and necroptosis. Second, no protocol for measuring mtDNA copy numbers was established. Hence, measuring the mtDNA copy number in serum, not in plasma, or using a different protocol might lead to different results. Third, this study was conducted at a single center in Korea, which might limit the generalization of our findings to other institutions or ethnic groups.

## 5. Conclusions

Plasma mtDNA copy number was determined to be a predictive factor of mortality in critically ill patients with sepsis and was strongly correlated with essential necroptosis mediators, suggesting that mtDNA propagates necroptosis and increases sepsis mortality.

## Figures and Tables

**Table 1:** Characteristics of the study population. Data are presented as number (percentage) or median (interquartile range).

	Total (n = 142)	Non-Survivors (n = 36)	Survivors (n = 106)	*p*-Value
Age, years	67 (53–74)	67 (52–72)	67 (54–75)	0.429
Male	95 (66.9)	27 (75.0)	68 (64.2)	0.232
Comorbidities				
All malignancies	75 (52.8)	22 (61.1)	53 (50.0)	0.249
Solid organ malignancies	51 (35.9)	16 (44.4)	35 (33.0)	0.217
Hematologic malignancies	25 (17.6)	6 (16.7)	19 (17.9)	0.864
Diabetes mellitus	43 (30.3)	12 (33.3)	31 (29.3)	0.645
COPD	11 (7.8)	5 (13.9)	6 (5.7)	0.146
Chronic kidney disease	11 (7.8)	2 (5.6)	9 (8.5)	0.730
Myocardial infarction	7 (4.9)	2 (5.6)	5 (4.7)	1.0
Congestive heart failure	5 (3.5)	2 (5.6)	3 (2.8)	0.601
Cerebrovascular disease	6 (4.2)	2 (5.6)	4 (3.8)	0.643
Chronic liver disease	13 (9.2)	6 (16.7)	7 (6.6)	0.070
Charlson Comorbidity Index	2 (1–3)	2 (2–3)	2 (1–3)	0.054
Clinical status on				
ICU admission				
Mechanical ventilation	57 (40.1)	20 (55.6)	37 (34.9)	0.029
Vasopressor support	49 (34.5)	17 (47.2)	32 (30.2)	0.063
Laboratory findings				
PaO2/FiO2 ratio	184 (126–281)	161 (121–245)	193 (127–285)	0.178
CRP, mg/dL	12.9 (5.0–23.0)	16.4 (7.7–25.0)	12.4 (3.9–21.8)	0.173
Lactate, mg/dL	2.7 (1.8–3.9)	3.3 (1.8–5.2)	2.7 (1.8–3.4)	0.151
Severity of illness				
SAPS 3 score	53 (46–59)	57 (48–71)	52 (45–58)	0.042
APACHE II score	23 (19–29)	26 (21–33)	22 (17–27)	0.004
SOFA score, initial	9 (6–11)	10 (7–12)	8 (6–11)	0.054

APACHE II, Acute Physiology and Chronic Health Evaluation II; COPD, chronic obstructive pulmonary disease; CRP, C-reactive protein; ICU, intensive care unit; PaO_2_/FiO_2_ ratio, ratio of arterial oxygen pressure to fractional inspired oxygen; SAPS 3, Simplified Acute Physiology Score 3; SOFA, Sequential Organ Failure Assessment.

**Table 2:** Plasma levels of mtDNA, RIPK3, MLKL, and HMGB in non-survivors versus survivors among critically ill patients with sepsis. Data are presented as median (interquartile range).

	Total (n = 142)	Non-Survivors (n = 36)	Survivors (n = 106)	*p*-Value
mtDNA, copies/µL	3090 (2015–4629)	4040 (3232–6288)	2585 (1867–3642)	<0.001
Necroptosis-related markers				
RIPK3, pg/mL	720 (259–1368)	1104 (512–2515)	536 (220–1197)	0.002
MLKL, ng/mL	2.4 (2.1–3.7)	3.2 (2.3–4.8)	2.3 (2.0–3.2)	0.004
HMGB1, ng/mL	5.0 (2.6–7.6)	6.8 (3.1–9.6)	4.5 (2.5–7.0)	0.017

RIPK3, receptor-interacting protein kinase-3; MLKL, mixed lineage domain-like pseudokinase; HMGB1, high-mobility group box 1.

## Data Availability

The data that support the findings of this study are available on request from the corresponding author. The data are not publicly available due to privacy or ethical restrictions.

## References

[1] Singer M., Deutschman C.S., Seymour C.W., Shankar-Hari M., Annane D., Bauer M., Bellomo R., Bernard G.R., Chiche J.D., Coopersmith C.M. (2016). The Third International Consensus Definitions for Sepsis and Septic Shock (Sepsis-3). JAMA.

[2] Fleischmann C., Scherag A., Adhikari N.K., Hartog C.S., Tsaganos T., Schlattmann P., Angus D.C., Reinhart K., International Forum of Acute Care T. (2016). Assessment of Global Incidence and Mortality of Hospital-treated Sepsis. Current Estimates and Limitations. Am. J. Respir. Crit. Care Med..

[3] Fleischmann-Struzek C., Mellhammar L., Rose N., Cassini A., Rudd K.E., Schlattmann P., Allegranzi B., Reinhart K. (2020). Incidence and mortality of hospital- and ICU-treated sepsis: Results from an updated and expanded systematic review and meta-analysis. Intensive Care Med..

[4] Marshall J.C., Reinhart K., International Sepsis F. (2009). Biomarkers of sepsis. Crit. Care Med..

[5] Yan H.P., Li M., Lu X.L., Zhu Y.M., Ou-Yang W.X., Xiao Z.H., Qiu J., Li S.J. (2018). Use of plasma mitochondrial DNA levels for determining disease severity and prognosis in pediatric sepsis: A case control study. BMC Pediatr..

[6] Harrington J.S., Choi A.M.K., Nakahira K. (2017). Mitochondrial DNA in Sepsis. Curr. Opin. Crit. Care.

[7] Bhagirath V.C., Dwivedi D.J., Liaw P.C. (2015). Comparison of the Proinflammatory and Procoagulant Properties of Nuclear, Mitochondrial, and Bacterial DNA. Shock.

[8] Timmermans K., Kox M., Scheffer G.J., Pickkers P. (2016). Plasma Nuclear and Mitochondrial DNA Levels, and Markers of Inflammation, Shock, and Organ Damage in Patients with Septic Shock. Shock.

[9] Schafer S.T., Franken L., Adamzik M., Schumak B., Scherag A., Engler A., Schonborn N., Walden J., Koch S., Baba H.A. (2016). Mitochondrial DNA: An Endogenous Trigger for Immune Paralysis. Anesthesiology.

[10] Mao J.Y., Li D.K., Zhang H.M., Wang X.T., Liu D.W. (2021). Plasma mitochondrial DNA levels are associated with acute lung injury and mortality in septic patients. BMC Pulm. Med..

[11] West A.P., Shadel G.S. (2017). Mitochondrial DNA in innate immune responses and inflammatory pathology. Nat. Rev. Immunol..

[12] Zhang Q., Raoof M., Chen Y., Sumi Y., Sursal T., Junger W., Brohi K., Itagaki K., Hauser C.J. (2010). Circulating mitochondrial DAMPs cause inflammatory responses to injury. Nature.

[13] Cheng Z., Abrams S.T., Toh J., Wang S.S., Wang Z., Yu Q., Yu W., Toh C.H., Wang G. (2020). The Critical Roles and Mechanisms of Immune Cell Death in Sepsis. Front. Immunol..

[14] Choi M.E., Price D.R., Ryter S.W., Choi A.M.K. (2019). Necroptosis: A crucial pathogenic mediator of human disease. JCI Insight.

[15] Yoo H., Im Y., Ko R.E., Lee J.Y., Park J., Jeon K. (2021). Association of plasma level of high-mobility group box-1 with necroptosis and sepsis outcomes. Sci. Rep..

[16] Yoo H., Lee J.Y., Park J., Yang J.H., Suh G.Y., Jeon K. (2020). Association of Plasma Level of TNF-Related Apoptosis-Inducing Ligand with Severity and Outcome of Sepsis. J. Clin. Med..

[17] Ma K.C., Schenck E.J., Siempos I.I., Cloonan S.M., Finkelsztein E.J., Pabon M.A., Oromendia C., Ballman K.V., Baron R.M., Fredenburgh L.E. (2018). Circulating RIPK3 levels are associated with mortality and organ failure during critical illness. JCI Insight.

[18] Mangalmurti N., Qing D., Hotz M., Siegel D.L., Sondheimer N., Mangalmurti N.S. (2016). Mitochondrial DNA Released Following Necroptosis Accumulates on RBCs. B97. Mitochondria Dysfunction in the Development of Lung Disease.

[19] Park J., Pabon M., Choi A.M.K., Siempos I.I., Fredenburgh L.E., Baron R.M., Jeon K., Chung C.R., Yang J.H., Park C.M. (2017). Plasma surfactant protein-D as a diagnostic biomarker for acute respiratory distress syndrome: Validation in US and Korean cohorts. BMC Pulm. Med..

[20] Knaus W.A., Draper E.A., Wagner D.P., Zimmerman J.E. (1985). APACHE II: A severity of disease classification system. Crit. Care Med..

[21] Moreno R.P., Metnitz P.G., Almeida E., Jordan B., Bauer P., Campos R.A., Iapichino G., Edbrooke D., Capuzzo M., Le Gall J.R. (2005). SAPS 3—From evaluation of the patient to evaluation of the intensive care unit. Part 2: Development of a prognostic model for hospital mortality at ICU admission. Intensive Care Med..

[22] Vincent J.L., Moreno R., Takala J., Willatts S., De Mendonca A., Bruining H., Reinhart C.K., Suter P.M., Thijs L.G. (1996). The SOFA (Sepsis-related Organ Failure Assessment) score to describe organ dysfunction/failure. On behalf of the Working Group on Sepsis-Related Problems of the European Society of Intensive Care Medicine. Intensive Care Med..

[23] Nakahira K., Kyung S.Y., Rogers A.J., Gazourian L., Youn S., Massaro A.F., Quintana C., Osorio J.C., Wang Z., Zhao Y. (2013). Circulating mitochondrial DNA in patients in the ICU as a marker of mortality: Derivation and validation. PLoS Med..

[24] Puskarich M.A., Shapiro N.I., Trzeciak S., Kline J.A., Jones A.E. (2012). Plasma levels of mitochondrial DNA in patients presenting to the emergency department with sepsis. Shock.

[25] Harrington J.S., Huh J.W., Schenck E.J., Nakahira K., Siempos I.I., Choi A.M.K. (2019). Circulating Mitochondrial DNA as Predictor of Mortality in Critically Ill Patients: A Systematic Review of Clinical Studies. Chest.

[26] Jung S.S., Moon J.S., Xu J.F., Ifedigbo E., Ryter S.W., Choi A.M., Nakahira K. (2015). Carbon monoxide negatively regulates NLRP3 inflammasome activation in macrophages. Am. J. Physiol. Lung Cell Mol. Physiol..

[27] Won J.H., Park S., Hong S., Son S., Yu J.W. (2015). Rotenone-induced Impairment of Mitochondrial Electron Transport Chain Confers a Selective Priming Signal for NLRP3 Inflammasome Activation. J. Biol. Chem.

[28] West A.P., Khoury-Hanold W., Staron M., Tal M.C., Pineda C.M., Lang S.M., Bestwick M., Duguay B.A., Raimundo N., MacDuff D.A. (2015). Mitochondrial DNA stress primes the antiviral innate immune response. Nature.

[29] Moreno-Gonzalez G., Vandenabeele P., Krysko D.V. (2016). Necroptosis: A Novel Cell Death Modality and Its Potential Relevance for Critical Care Medicine. Am. J. Respir. Crit. Care Med..

[30] Linkermann A., Green D.R. (2014). Necroptosis. N. Engl. J. Med..

[31] He S., Wang X. (2018). RIP kinases as modulators of inflammation and immunity. Nat. Immunol..

[32] Kaczmarek A., Vandenabeele P., Krysko D.V. (2013). Necroptosis: The release of damage-associated molecular patterns and its physiological relevance. Immunity.

[33] Kaiser W.J., Sridharan H., Huang C., Mandal P., Upton J.W., Gough P.J., Sehon C.A., Marquis R.W., Bertin J., Mocarski E.S. (2013). Toll-like receptor 3-mediated necrosis via TRIF, RIP3, and MLKL. J. Biol. Chem..

[34] Takaoka A., Wang Z., Choi M.K., Yanai H., Negishi H., Ban T., Lu Y., Miyagishi M., Kodama T., Honda K. (2007). DAI (DLM-1/ZBP1) is a cytosolic DNA sensor and an activator of innate immune response. Nature.

